# Biosynthesized chitosan nanoparticles from Krameria lappacea: a strategy to ameliorate coccidiosis-induced intestinal dysfunction

**DOI:** 10.3389/fcimb.2026.1915430

**Published:** 2026-09-03

**Authors:** Mohammed I. Alquraishi, Esam M. Al-Shaebi, Chinnadurai Veeramani, Ahmed S. El Newehy, Khalid S. Al-Numair, Mohammed A. Alsaif, Saleh Al Quraishy, Rewaida Abdel-Gaber

**Affiliations:** 1 Department of Community Health Sciences, College of Applied Medical Sciences, King Saud University, Riyadh, Saudi Arabia; 2 Department of Zoology, College of Science, King Saud University, Riyadh, Saudi Arabia

**Keywords:** anticoccidial activity, chitosan nanoparticles, *Eimeria papillata*, *Krameria lappacea*, *Murine coccidiosis*, oxidative Stress

## Abstract

**Background:**

Coccidiosis is a major parasitic disease caused by *Eimeria* species that induces intestinal damage, oxidative stress, and impaired nutrient absorption. The risk associated with drug-resistant strains and other adverse effects related to conventional anticoccidial drugs necessitates the development of safer natural alternatives. Thus, in this study, we examined the anticoccidial efficacy of biosynthesized chitosan nanoparticles (CNPs) derived from *Krameria lappacea* root extract against *Eimeria papillata*-induced murine coccidiosis.

**Methods:**

Chitosan nanoparticles were biosynthesized using *K. lappacea* root extract and characterized by transmission electron microscopy (TEM), UV–visible spectroscopy, and Fourier-transform infrared spectroscopy (FT-IR). Male C57BL/6 mice were experimentally infected with 10³ sporulated oocysts of *E. papillata* and treated orally with CNPs at doses of 5, 10, and 20 mg/kg, while amprolium (120 mg/kg) was used as a reference coccidian drug. Fecal oocyst output, body weight changes, jejunal histopathology, intracellular parasitic stages, oxidative stress biomarkers, antioxidant enzyme activities, glucose, and total protein contents were evaluated on day five post-infection.

**Results:**

TEM analysis revealed that the synthesized CNPs were predominantly spherical with particle sizes ranging from 57.01 to 83.05 nm with an average diameter of about 70.16 nm. FT-IR analysis indicated the presence of functional groups associated with alcohols, amines, sulfates, and halo compounds. Infection with *E. papillata* caused significant body weight loss, increased oocyst shedding, severe jejunal histopathological alterations, elevated nitric oxide (NO) and hydrogen peroxide (H_2_O_2_) levels, and reduced the levels of glucose, total protein, catalase (CAT), and glutathione peroxidase (GPx). Treatment with CNPs significantly reduced fecal oocyst shedding in all treated groups; however, the 5 mg/kg dose was the most effective, lowering oocyst output to 1.308 × 10^6^ ± 2.54 × 10^5^ oocysts/g feces compared with 7.196 × 10^6^ ± 2.77 × 10^5^ oocysts/g feces in infected untreated mice. The reductions achieved with the 10 and 20 mg/kg CNP doses were less pronounced. CNPs administration also markedly decreased intracellular parasitic stages, improved jejunal histological architecture, restored glucose and protein contents, promoted antioxidant capacity, and lowered oxidative stress. The therapeutic efficacy of CNPs was comparable to or superior to that of the reference drug, amprolium.

**Conclusion:**

CNPs exhibited potent anticoccidial, antioxidant, and protective effects against *E. papillata* infection in mice. These findings suggest that CNPs are a promising natural alternative for the management of coccidiosis, warranting further investigation into their mechanisms of action, safety, and potential applications in veterinary medicine.

## Introduction

Coccidiosis is a critical parasitic disease affecting both domestic and laboratory animals worldwide (Mesa-Pineda et al., 2021; Razavi et al., 2024). Indeed, coccidiosis has increased the economic burden of animal production, especially in the poultry and cattle industries, posing a potential threat to global food security (Blake and Tomley, 2014; Pal and Tariku, 2024). Coccidiosis is mainly caused by protozoan parasites belonging to the genus *Eimeria*, which invade the intestinal epithelium to induce severe pathological alterations, including villous atrophy, inflammatory infiltration, oxidative stress, diarrhea, malabsorption, and impaired growth performance (Mathis et al., 2024; Chen et al., 2025). In murine models, *Eimeria papillata* infection is widely utilized to investigate host–parasite interactions and evaluate novel anticoccidial agents due to its well-characterized life cycle and reproducible intestinal pathology (Dkhil et al., 2011; Ismaeil et al., 2025). Despite the extensive use of conventional anticoccidial drugs, including amprolium and sulfonamides, their prolonged administration has resulted in the emergence of drug-resistant strains, drug residues in animal products, and potential adverse effects on host tissues (Chalalai et al., 2025). Therefore, it is critical to develop safer and more effective alternative therapeutic strategies derived from natural sources (Flores et al., 2026).

Recently, medicinal plants have attracted increasing attention in the research field as a rich source of bioactive compounds with antiparasitic, antioxidant, anti-inflammatory, and immunomodulatory properties (Saeed and Alkheraije, 2023). Among these plants is *Krameria lappacea*, which has been traditionally used to treat various infectious and inflammatory diseases (Abdel-Gaber et al., 2025). The roots of *K. lappacea* contain a diverse range of bioactive constituents, including phenolics, flavonoids, tannins, and lignans, which exert potent biological activities, including antimicrobial, antioxidant, and antiparasitic effects (Abdel-Gaber et al., 2024). Previous phytochemical studies have demonstrated that extracts of *K. lappacea* possess significant radical scavenging activity and inhibitory effects against several pathogenic microorganisms, suggesting their potential application in controlling parasitic infections (Zabka, 2022; Abdel-Gaber et al., 2024, 2025). Together, these studies highlighted the potency of *K. lappacea* as a potential agent against coccidiosis that warrants further investigation.

In recent years, the application of nanotechnology has offered a potential approach to enhance the therapeutic efficacy and bioavailability of natural compounds ( (Sanjai et al., 2024). Chitosan nanoparticles have attracted considerable interest in biomedical and pharmaceutical industries partly due to their biocompatibility, biodegradability, low toxicity, and remarkable antimicrobial properties (Zhao et al., 2018; Ul-Islam et al., 2023). Chitosan, a naturally occurring polysaccharide derived from chitin, can enhance drug delivery, elevate cellular uptake, and prolong the retention time of bioactive constituents at target sites (Cheung et al., 2015; Alquraishi et al., 2025). Moreover, biosynthesis of nanoparticles using plant extracts, commonly referred to as green synthesis, is considered eco-friendly and cost-effective compared to other nanoparticle synthesis methods (Filho et al., 2023; Edo et al., 2025). Plant-mediated biosynthesized nanoparticles are often enriched with phytochemicals that act as reducing and stabilizing agents, thereby enhancing their biological activities (Zuhrotun et al., 2023).

Several reports have indicated the antiparasitic potential of chitosan-based nanoparticles against protozoan and helminthic infections through mechanisms involving parasite membrane disruption, induction of oxidative stress for the host, inhibition of sporulation, and modulation of host immune responses (Gaafar et al., 2014; Firouzeh et al., 2021; Norouzi et al., 2024; Khedr et al., 2025). However, limited information is available regarding the anticoccidial efficacy of CNPs derived from *K. lappacea* root extract, particularly in experimental murine coccidiosis. Therefore, the current study was designed to investigate the anticoccidial potential of CNPs prepared using *K. lappacea* root extract against *E. papillata*-induced murine coccidiosis. In addition, this study aimed to evaluate their effects on parasitic species burden, intestinal histopathological alterations, and associated oxidative stress levels, to explore their potential as a natural alternative for the management of coccidian infections.

## Materials and methods

### Preparation of *Krameria lappacea* root extract

Roots of *K. lappacea* were obtained from a local herbal market in Riyadh, Saudi Arabia, and identified by a taxonomist at the Herbarium of the College of Science, King Saud University. A voucher sample was preserved in the herbarium collection under accession number KSU-22958. Preparation of the 70% methanolic root extract was carried out according to a previously published method (Manikandan et al., 2008). In brief, the roots were shade-dried at room temperature and processed with an electric grinder (Senses, MG-503T, Korea) to yield a fine powder. The powdered roots were then extracted with 70% methanol at room temperature under continuous stirring for 24 h, followed by filtration through Whatman No. 1 filter paper. Next, the filtrate was concentrated under reduced pressure using a rotary evaporator (Buchi, Switzerland) at 50 °C, then allowed the filtrate to dry to yield the crude extract. Finally, we stored the extract at 4 °C until further use for the biosynthesis of chitosan nanoparticles (CNPs).

### Biosynthesis of chitosan nanoparticles

For chitosan biosynthesis, we followed a previously published protocol of El-Naggar et al. with minor modifications (El-Naggar et al., 2022). In brief, medium-molecular-weight chitosan (190–310 kDa) with a degree of deacetylation of ≥75% and a purity of >90% was dissolved at 1% (w/v) in 0.1% acetic acid, and 1 N NaOH was used to adjust the pH to 4.6. The chitosan mixture solution was completely dissolved using a magnetic agitator for 1 h. 10 ml of the prepared plant extract (2.0 mg/ml) was added to the chitosan mixture solution under an agitator shaker, drop by drop, at room temperature until a clear, opalescent solution was obtained. Further, it was allowed to mix well under an agitator shaker for another 1 hour. The nanoparticle suspension was centrifuged at 15,000 × g for 30 min, and the obtained pellet was rinsed with 70% (v/v) ethanol and air-dried at 37 °C.

### Characterization of biosynthesized Chitosan nanoparticles (CNPs)

#### Transmission electron microscopy (TEM)

We confirmed the CNP synthesis by collecting reaction mixture aliquots at predetermined time intervals and recording their UV–visible absorption spectra using a spectrophotometer (UV-3600, Shimadzu). The morphology, surface architecture, and particle-size distribution of CNPs were analyzed by TEM. Before analysis, the nanoparticle suspension was ultrasonically dispersed to achieve homogeneous distribution, and a small aliquot was placed onto a carbon-coated copper grid and left to dry at room temperature. TEM imaging was performed with a high-resolution JEOL JEM-1011 transmission electron microscope (JEOL Ltd., Tokyo, Japan) at an accelerating voltage of 80 kV. The resulting micrographs demonstrated that the synthesized nanoparticles were predominantly spherical, uniformly distributed, and within the nanoscale range. Images were recorded at a magnification of 30000× with a scale bar of 200 nm.

#### Fourier transform infrared spectroscopy (FT-IR)

The functional groups and chemical characteristics of CNPs were analyzed using FT-IR. For sample preparation, the CNPs were thoroughly blended with potassium bromide (KBr) powder at a ratio of 1:99 (w/w) to produce a translucent disc according to the method described by Alkhudhayri et al. (2020). FT-IR analysis was performed using a NICOLET 6700 Fourier-transform infrared spectrometer (Thermo Scientific, Waltham, MA, USA). Spectral data were recorded over the wavelength range of 4000–400 cm^-^¹, and the characteristic absorption peaks were identified based on their maximum absorbance values.

### Experimental animals

Male C57BL/6 mice (weighing about 20–25 g and aged 9–12 weeks) were used in this study. The animals were obtained from the animal facility at the College of Pharmacy, King Saud University, Riyadh, Saudi Arabia. Mice were housed in polypropylene cages under standard laboratory conditions (including a controlled temperature of 22 ± 2 °C and relative humidity of 50–60%, with a 12 h light/dark photoperiod). Animals were acclimatized for 1 week before the initiation of the experiment and were provided with a standard commercial pellet diet and water ad libitum throughout the study.

### Parasite and experimental infection

In this study, *Eimeria papillata* was used as the model to induce coccidiosis, which has been morphometrically and molecularly characterized by Al-Quraishy et al. (2022). Briefly, five mice (*Mus musculus*) were infected with 10^3^ sporulated oocysts via gavage administration. Fecal samples were gathered five days after infection, and oocysts were isolated following the flotation method (Kumar et al., 2014). The recovered oocysts were maintained in a 2.5% (w/v) aqueous potassium dichromate solution (K_2_Cr_2_O_7_) under suitable conditions to allow sporulation and were subsequently rinsed in phosphate-buffered saline (PBS, pH 7.4) (Sigma Aldrich, Taufkirchen, Germany) for further experimental use.

### Experimental design

Following a one-week acclimatization period, male C57BL/6 mice were randomly allocated to seven experimental groups (n = 5 per group) using a simple randomization procedure, ensuring that each animal had an equal probability of assignment to any group. The groups were organized as follows: Group 1: Served as a negative control (no treatment), Group 2: Treated with CNPs only (5 mg/kg), Group 3: Served as a positive control, infected-non-treated group, Group 4 to 6: Mice were infected and treated with CNPs dosage of 5 mg/kg, 10 mg/kg, and 20 mg/kg, respectively, and Group 7: Infected and treated with the Amprolium (120 mg/kg), the reference drug. All groups, except groups 1 and 2, were orally inoculated with 10^3^ sporulated oocysts of *E. papillata*, as described by Abdel-Tawab et al. (2020). To evaluate the early therapeutic efficacy of CNPs, treatments were administered orally 60 min after parasite inoculation, thereby ensuring that the animals had already been exposed to the infective oocysts while targeting the initial stages of parasite invasion and intracellular development. The assigned treatments were administered once daily throughout the experimental period.

### Determination of oocyst output and body weight

During day five post-infection (p.i.), we collected fresh fecal pellets from all experimental groups to examine the presence of *Eimeria* oocysts using a McMaster chamber. All numbers of oocysts were expressed per gram (OPG) of wet feces following the method described by Dkhil et al. (2015). Body weights of all mice were recorded throughout the experimental period to evaluate the effects of infection and treatment, as described by Al-Quraishy et al. (2020).

### Sample collection

At the end of the experiment, all mice were humanely sacrificed by carbon dioxide (CO_2_) asphyxiation. The jejunal tissues were immediately excised, rinsed gently with cold saline solution, and divided into small portions for subsequent analysis. For histopathological examination, jejunal tissue samples were fixed in 10% neutral buffered formalin (NBF) for 24 hr at 27 °C. An additional jejunal samples were rapidly transferred into sterile microtubes and then stored at −80 °C for further analysis.

### Histopathological examination

The preserved jejunal samples were accordingly processed, embedded in paraffin, sectioned at 5 µm, and finally stained with hematoxylin and eosin (H&E) following the procedure described by Drury and Wallington (1980). Histopathological observations were conducted using a Leica DM 2500 light microscope, and NIS-Elements software (version 3.8) was used for microscopic imaging and detailed tissue analysis. Moreover, the developmental stages of *E. papillata* in the jejunal sections were quantified by counting parasites within 10 villus–crypt units (VCU).

### Biochemical analysis

Jejunal tissues were accurately weighed and disrupted in chilled Tris-HCl/sucrose buffer (50 mM Tris-HCl and 300 mM sucrose) to obtain a 10% (w/v) homogenate following a previously published protocol (Tsakiris et al., 2004). The prepared homogenates were centrifuged at 500 ×g for 10 min at 4 °C, and the collected supernatants were used for further assays. Biochemical parameters were determined colorimetrically using commercial diagnostic kits obtained from Biodiagnostic Co. (Egypt). Absorbance readings were measured using a Molecular Devices Spectra MAX 190 microplate reader equipped with SoftMax^®^ Pro software version 6.3.1.

Jejunal glucose concentrations were determined according to Trinder (1969) method, whereas the total protein levels were measured following the procedure of Gornall et al. (1949). The level of hydrogen peroxide (H_2_O_2_), as an indicator of oxidative stress, was evaluated in the jejunal homogenates using the method of Aebi (1984). Nitric oxide (NO) production was quantified based on the accumulation of nitrite according to the protocol of Green et al. (1982). Furthermore, the antioxidant enzyme activities of catalase (CAT) and glutathione peroxidase (GPx) were assessed using the methods of Aebi (1984) and Paglia and Valentine (1967), respectively.

### Statistical analysis

All experimental data are presented as the mean ± standard deviation (SD). Each individual mouse was considered the experimental unit for all statistical analyses. Prior to hypothesis testing, the assumptions of one-way analysis of variance (ANOVA), including normality of data distribution (Shapiro–Wilk test) and homogeneity of variance (Levene’s test), were evaluated. As the data satisfied these assumptions, statistical comparisons among the experimental groups were performed using one-way ANOVA, followed by Tukey’s multiple comparison *post hoc* test. Statistical analyses were conducted using IBM SPSS Statistics for Windows, Version 29.0 (IBM Corp., Armonk, NY, USA). Differences were considered statistically significant at *p* ≤ 0.05.

## Results

**Figure 1:**
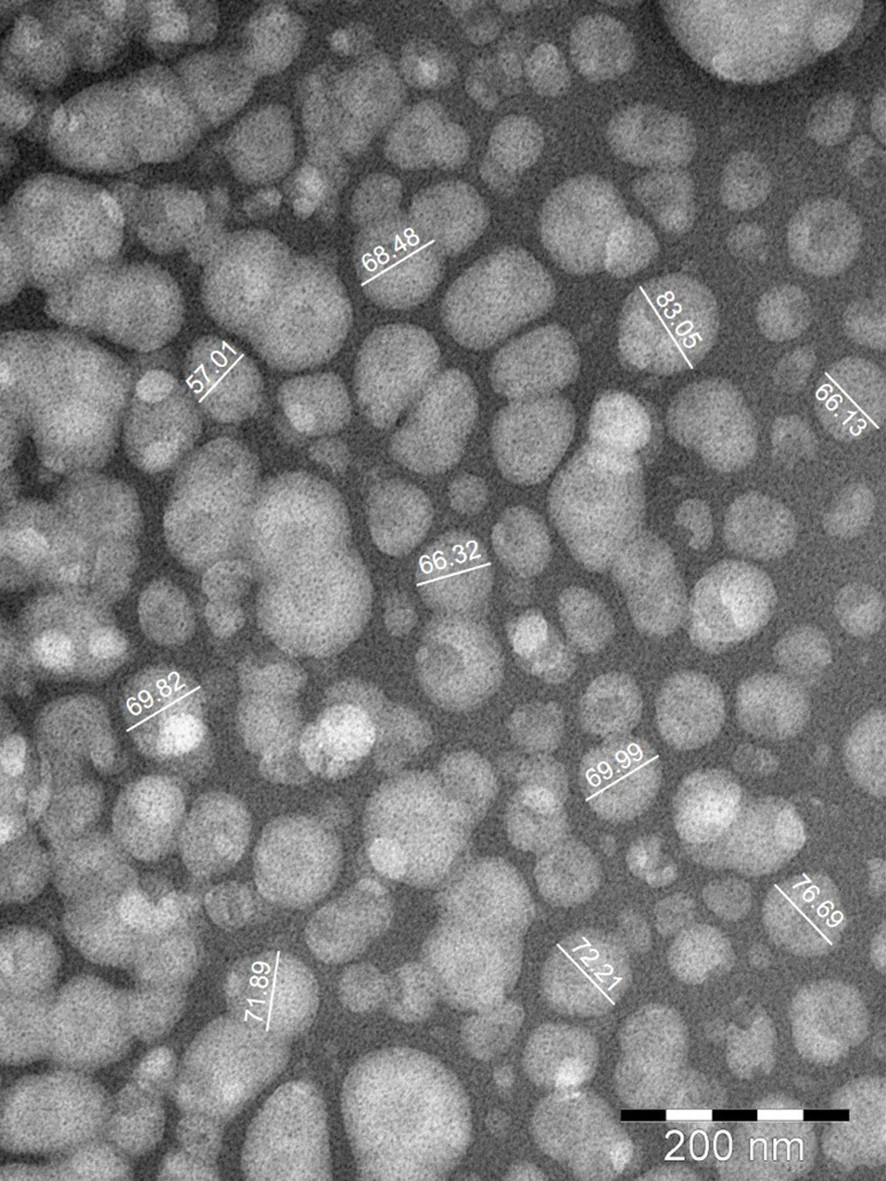
TEM of biosynthesized CNPs at a magnification of 30000× (scale bar= 200 nm).

**Figure 2:**
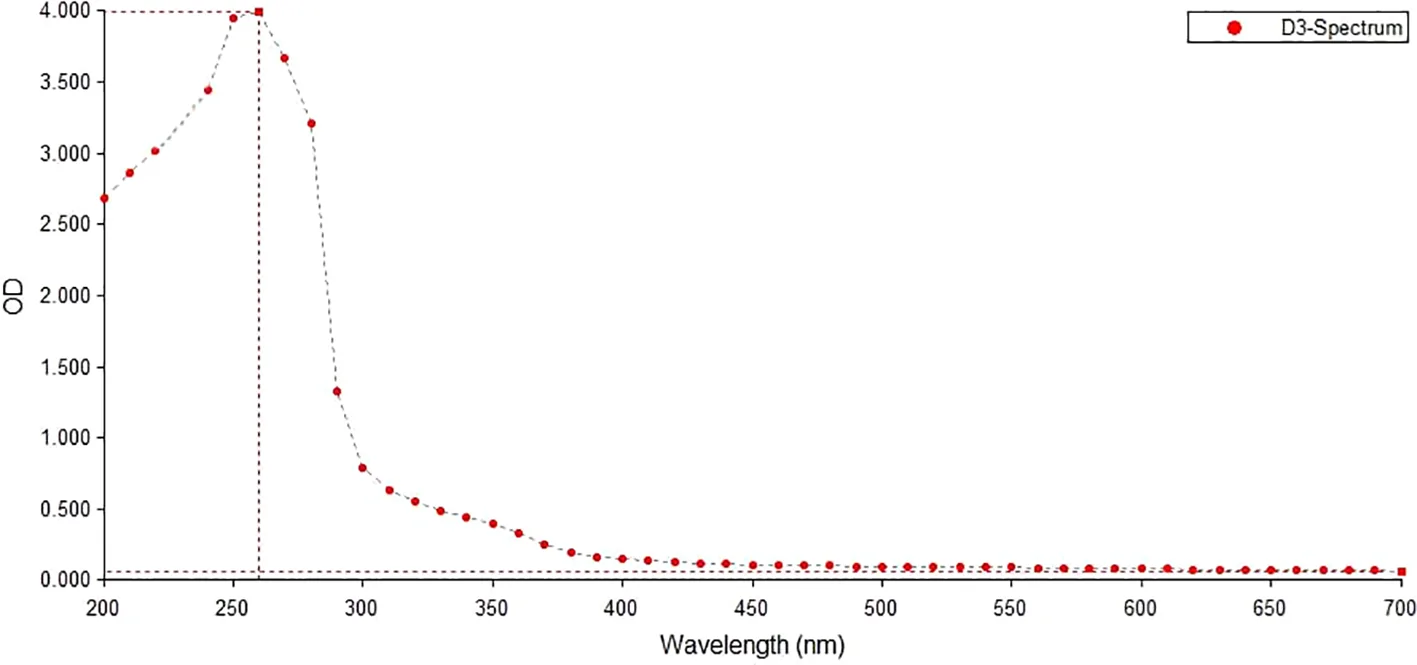
UV spectroscopic graph of biosynthesized CNPs showing absorbance.

The TEM method revealed CNP sizes ranging from 57.01 to 83.05 nm, with an average diameter of ∼70.16 nm, and spherical shapes (**Figure 1**). After 4 min of a reduction reaction, the UV-visible absorption spectra of CNPs showed a single peak at 260 nm (**Figure 2**). Together, these results confirmed the presence of CNP nanoparticles.

**Figure 3:**
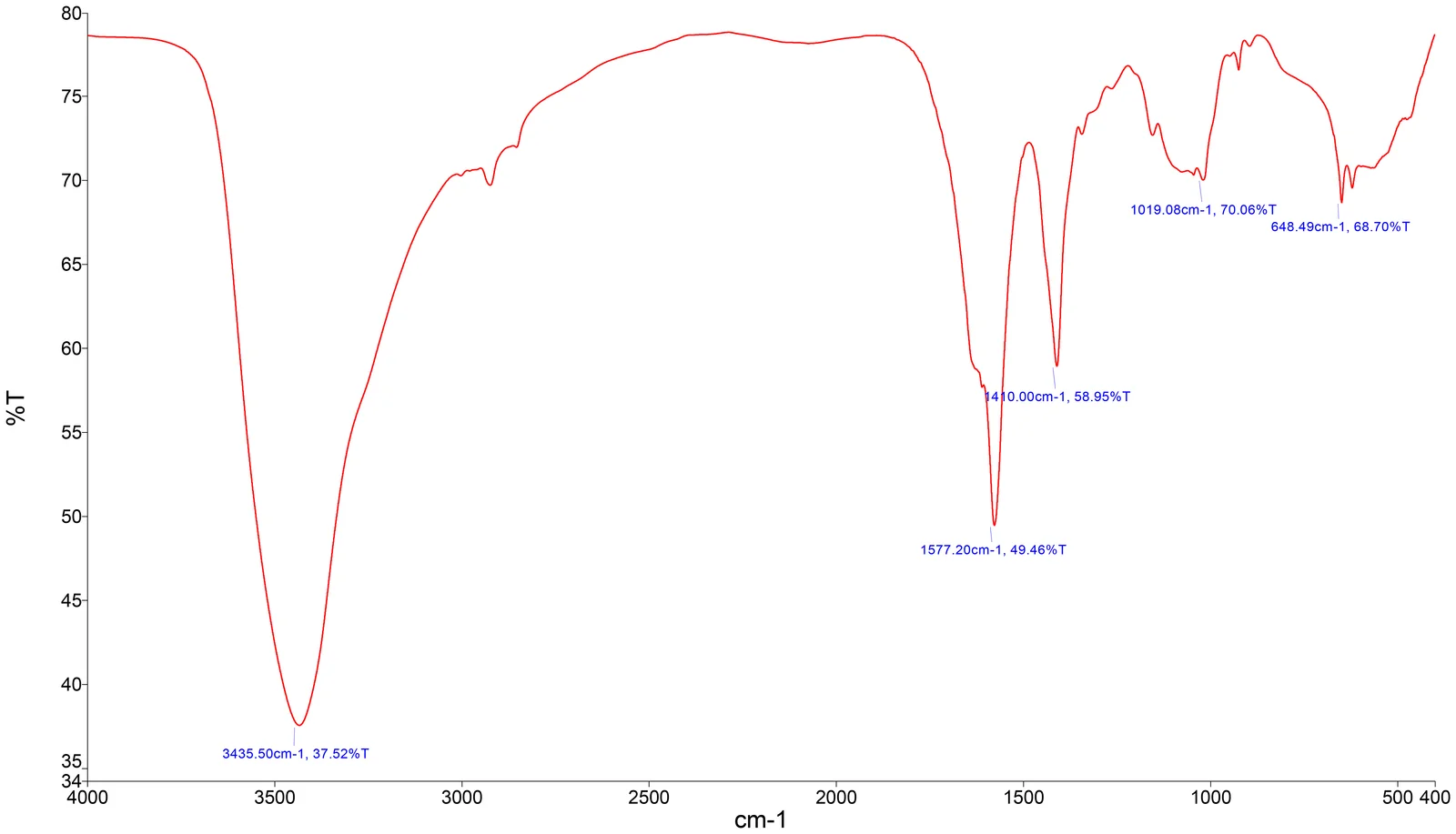
FT-IR of CNPs in an aqueous solution showing the functional properties of the material.

Further analysis of CNPs using FT-IR indicated various bands at 3435.50 cm^-1^, 1577.20 cm^-1^, 1410.00 cm^-1^, 1019.08 cm^-1^, and 648.49 cm^-1^, as shown in **Figure 3** and **Table 1**. O-H stretching was indicated by the band at 3435.50 cm^-1^, suggesting the potential presence of alcohol, while the band at 1577.20 cm^-1^ may reflect C=C stretching for the presence of a cyclic alkene. Moreover, S=O stretching at 1410.00 cm^-1^ indicates the presence of sulfate, and the band at 1019.08 cm^-1^ might correspond to C-N stretching for the presence of amine. Finally, C-Br stretching at 648.49 cm^-1^ may reflect the presence of a halo compound.

**Table 1:** FT-IR for CNPs.

Absorption (cm^-1^)	Transmittance (%)	Appearance	Group	Compound class
3435.50	13.18004	strong, broad	O-H stretching	alcohol
1577.20	17.37184	medium	C=C stretching	cyclic alkene
1410.00	20.70382	strong	S=O stretching	sulfate
1019.08	24.60641	medium	C-N stretching	amine
648.49	24.13789	strong	C-Br stretching	halo compound

**Figure 4:**
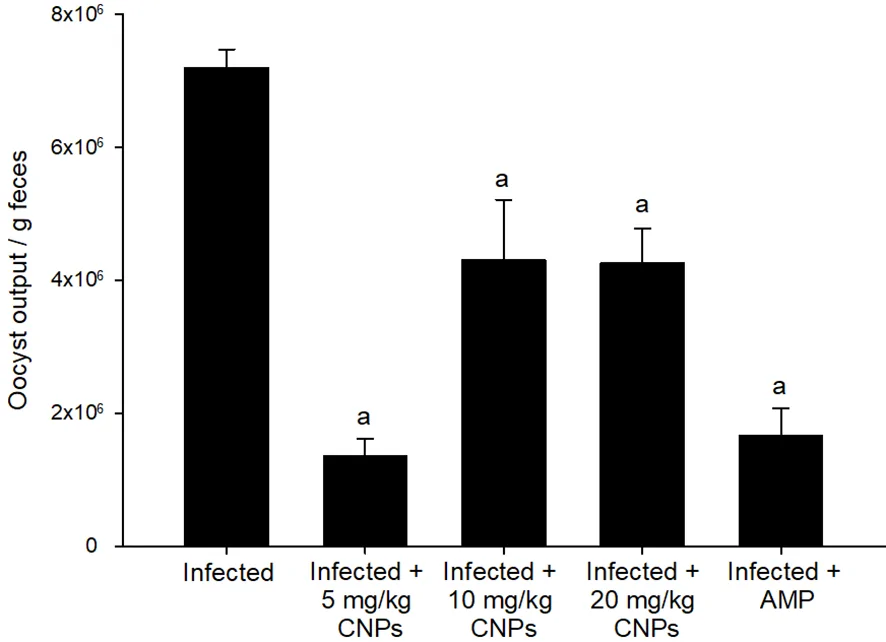
Oocyst output in mice infected with *Eimeria papillata* and the infected treated groups with 5 mg/kg, 10 mg/kg, 20 mg/kg CNPs, and 120 mg/kg AMP. All values are represented as means ± SD. ^a^indicate the significance (p ≤ 0.05) between the infected and treatment groups.

**Figure 5:**
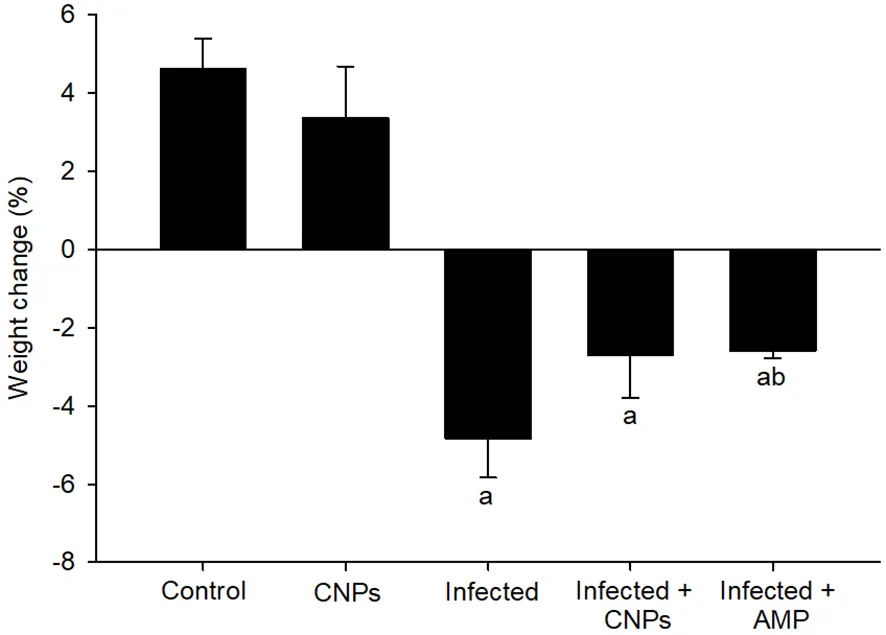
Effect of CNPs on the body weight of mice after infection with *Eimeria papillata*. Values are represented as means ± SD. ^a^indicates significance (p ≤ 0.05) against the control, ^b^indicates significance (p ≤ 0.05) against infected group.

Regarding oocyst output, there was significant variation between experimental groups on day five post-infection (p.i.) (**Figure 4**). In the infected group, the number of oocysts shedding was approximately 7.196 × 10^6^ ± 2.77 × 10^5^ per g of feces. However, this number was significantly reduced after CNPs treatment, with an approximate oocyst output of 1.308 × 10^6^ ± 1.36 × 10^5^ per g of feces observed in the group treated with 5 mg/kg. Yet, this reduction was less significant in the groups treated with 10 and 20 mg/kg. The dose of 5 mg/kg was considered the most effective compared with the reference drug (1.66 × 10^6^ ± 4.05 × 10^5^ oocysts/g feces) and was thus used for further analysis (**Figure 4**). Regarding mouse growth, the negative control group showed normal growth, with an average weight gain of about 4.63% (**Figure 5**). However, after infection with *E. papillata*, the mice had lost about 4.83% of their original weight, and the treatment with CNPs (5 mg/kg) reduced the weight loss to about -2.69% (**Figure 5**).

**Figure 6:**
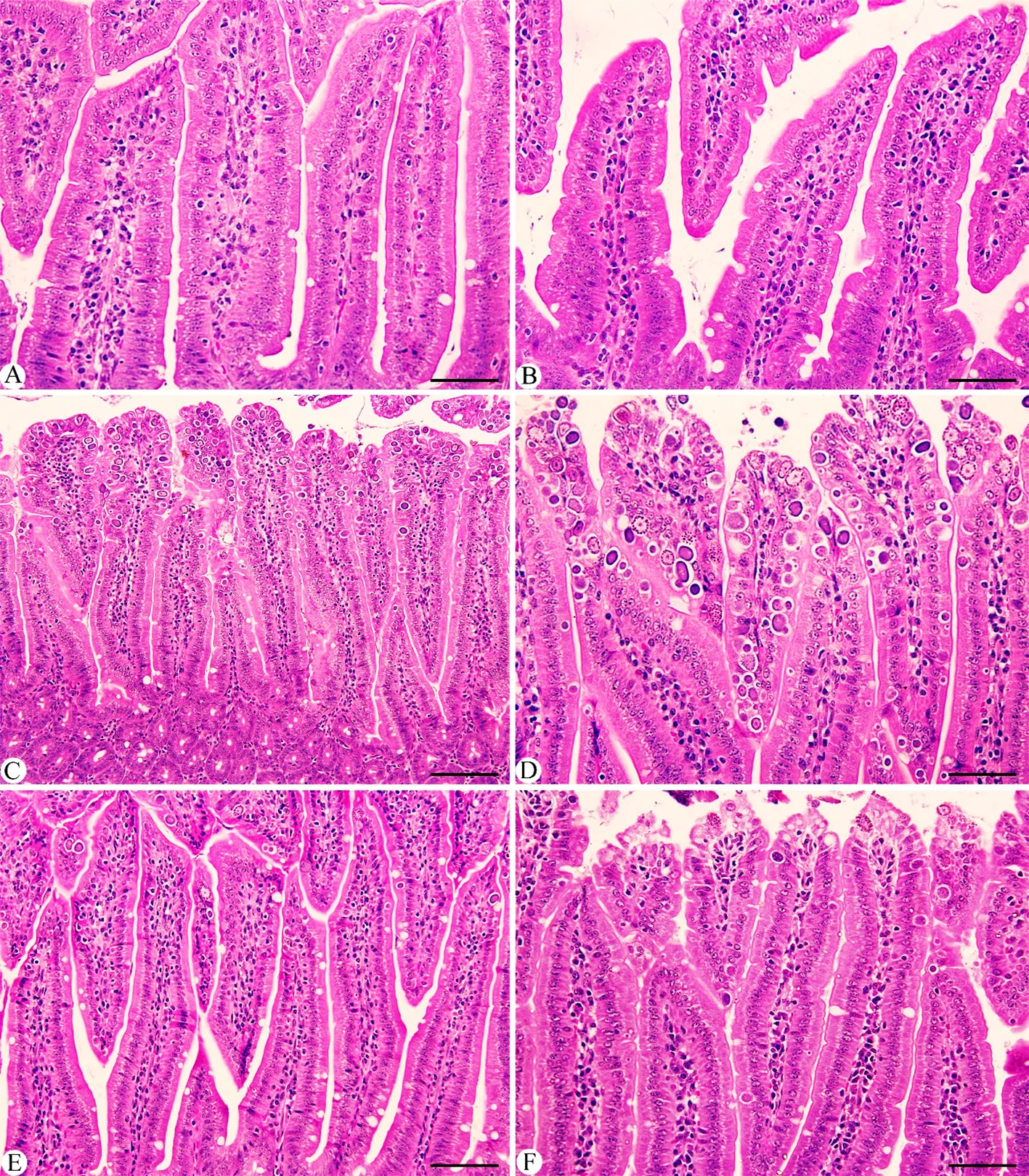
Histology of the mice jejunum for experimental groups. **(A)** Control group. **(B)** Non-infected-treated group with CNPs. **(C–F)** Infected groups with sporulated *E. papillata* as follows: **(C, D)** Infected group only. **(E)** Infected-treated group with CNPs. **(F)** Infected-treated group with AMP. Scale bar = 50 µm.

**Figure 7:**
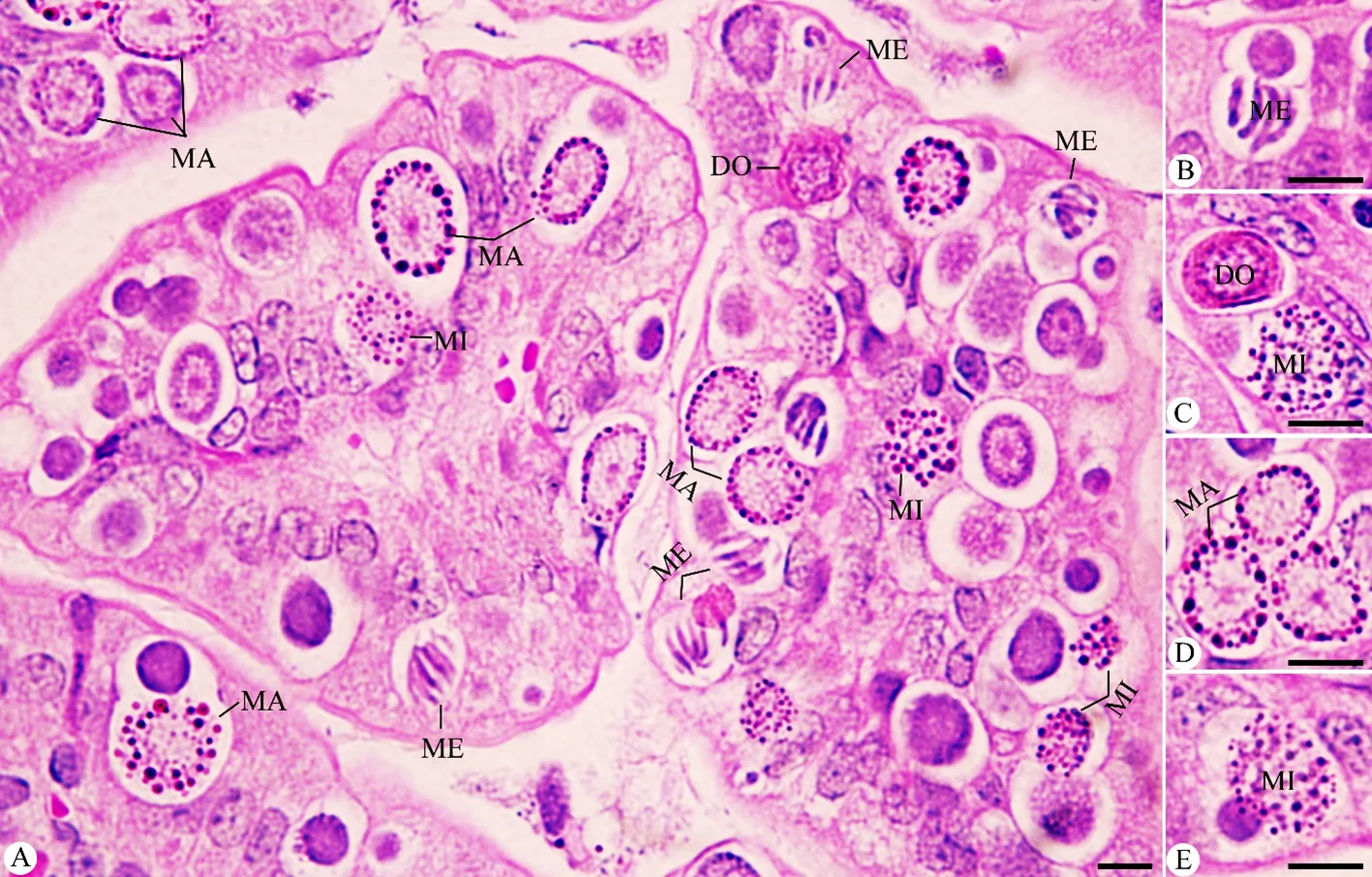
**(A)** Sections of the infected jejunum stained with hematoxylin and eosin (H&E) on day five p.i., indicating the different developmental stages. Meronts (ME) are shown in **(B)**; microgamonts (MI) in **(C, E)**, macrogamonts (MA) in **(D)**, and developing oocysts (DO) in **(C)**. Scale bar= 10 µm.

**Figure 8:**
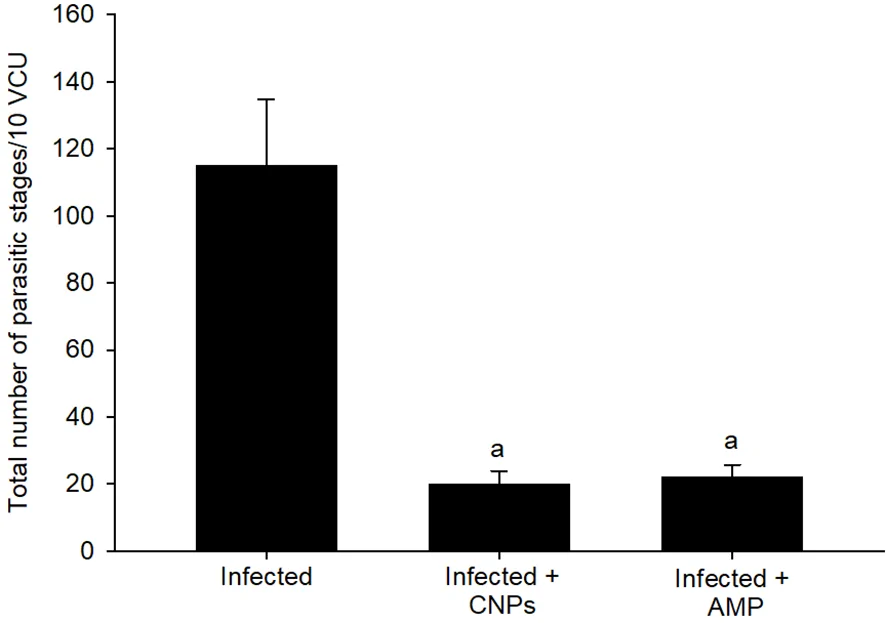
Treatment with CNPs and AMP induced significant changes in the overall number of parasitic stages of *Eimeria papillata* in jejunum tissue per 10 VCU on day five p.i. All values are represented as means ± SD. ^a^indicates significance (p ≤ 0.05) compared to the infected group.

Next, we examined *Eimeria* developmental stages in epithelial jejunum using light microscopy and hematoxylin/eosin staining. The presence of developmental stages has been confirmed in experimental groups, as shown in **Figure 6**. Moreover, **Figure 7** revealed the development of meronts, microgamonts, macrogamonts, and developing oocysts in the intestinal epithelium of the infected groups. Importantly, the number of intracellular parasitic stages was significantly decreased when mice were treated with CNPs, from 114.96 ± 19.69 in the infected group to 19.90 ± 3.94 stages/10 VCU (**Figure 8**).

**Figure 9:**
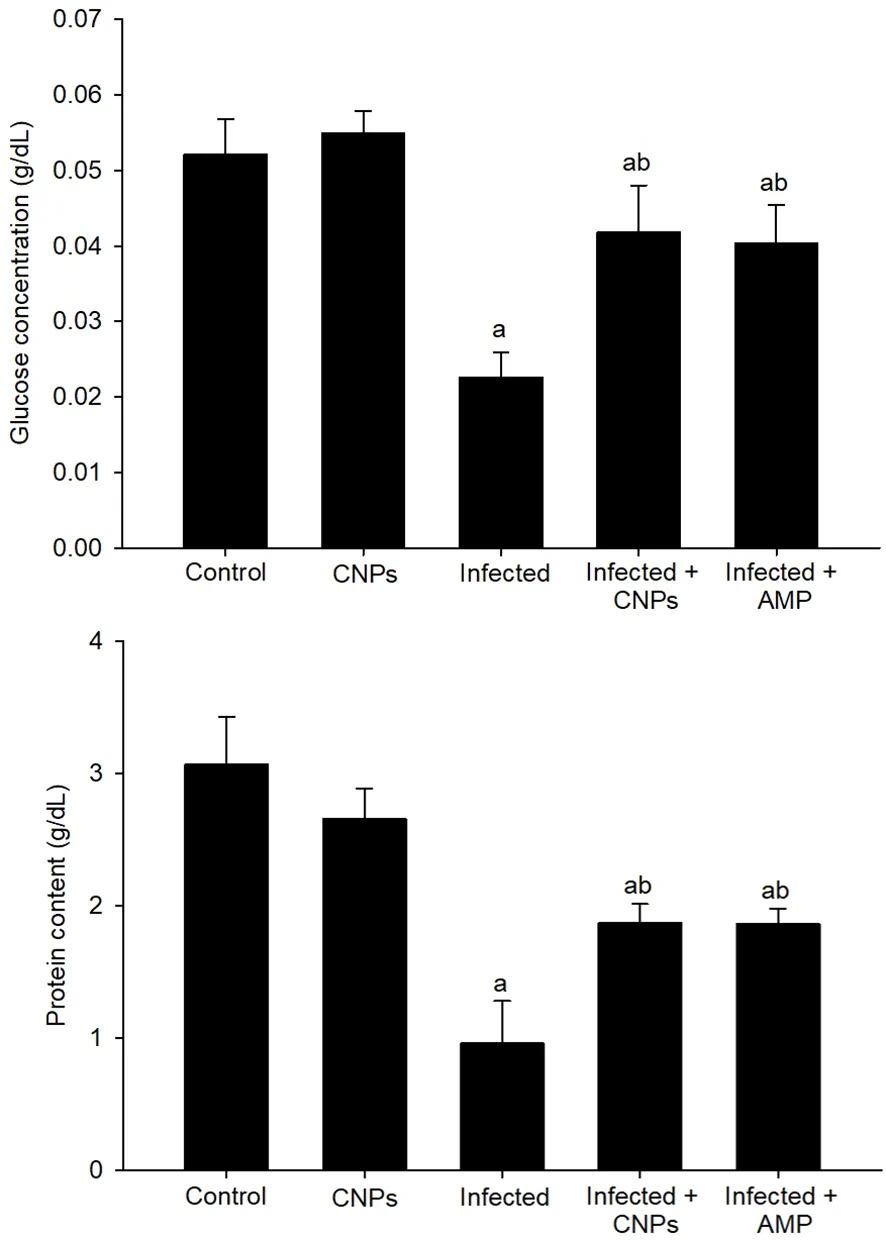
Effects of CNPs on the glucose and protein contents in the jejunal tissue of mice infected with *E. papillata*. ^a^Indicates (p ≤ 0.05) significant change compared to the control, ^b^(p ≤ 0.05) indicates significant change compared to the infected group.

As shown in **Figure 9**, nutrient constituents were detected in the mice jejunum. Quantitative analysis revealed a depletion in glucose levels in mice jejuna infected with *E. papillata* (0.022 ± 0.003 g/dL) compared to the control group (0.052 ± 0.004 g/dL; **Figure 9**). Oral administration of CNPs caused a significant elevation in glucose levels (0.041 ± 0.006 g/dL) compared to the infected group (**Figure 9**). Moreover, the *E. papillata* infection reduced total protein content in the mice’s jejunum (0.95 ± 0.32 g/dL) compared with the control (3.07 ± 0.36 g/dL) (**Figure 9**). However, treatment with CNPs restored protein content (1.87 ± 0.13 g/dL) in the jejunum compared with the infected group (**Figure 9**).

**Figure 10:**
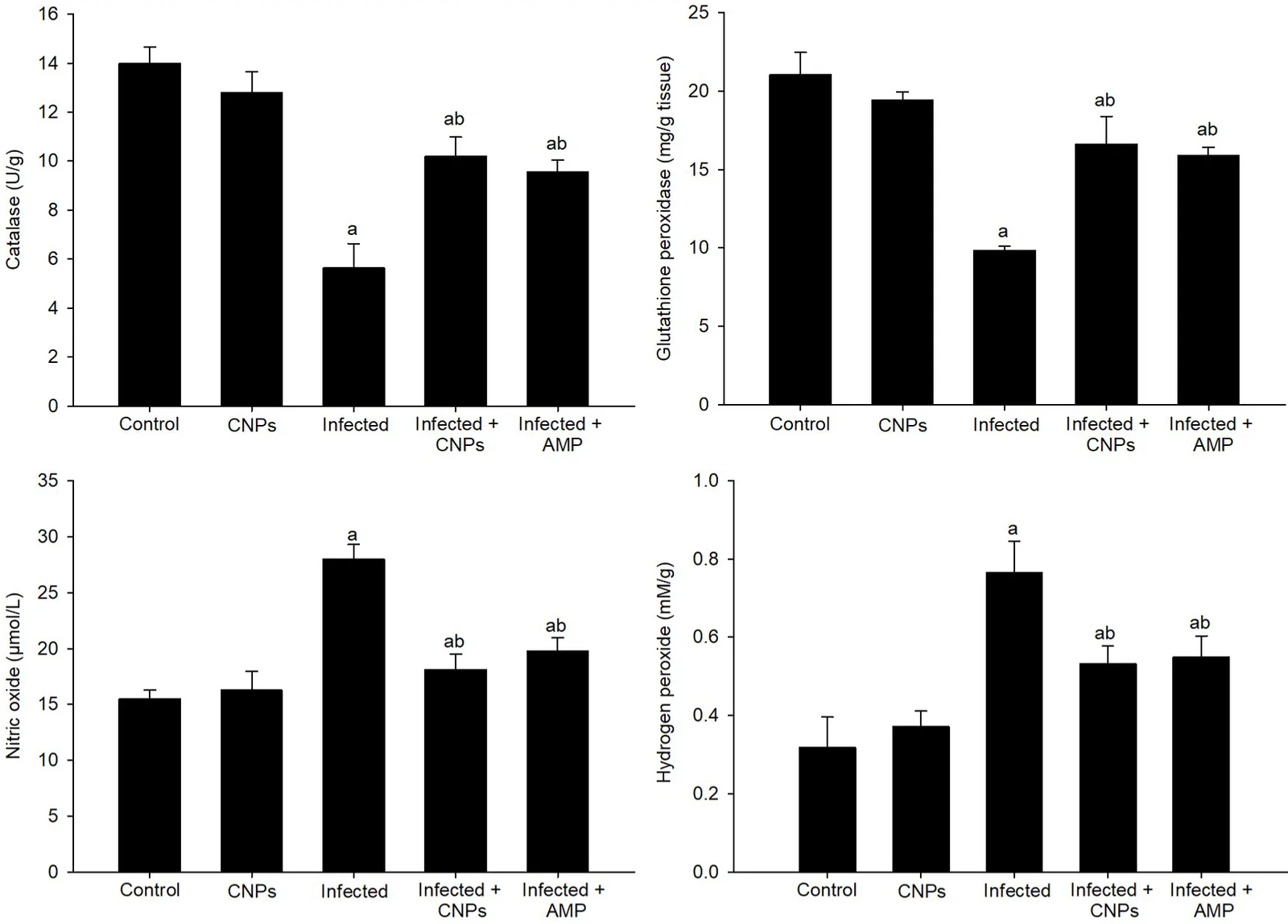
Protective effects of CNPs on the level of Catalase (CAT), glutathione peroxidase (GPx), nitric oxide (NO), and hydrogen peroxide (H_2_O_2_) levels of the mice jejunum infected with *E. papillata*. ^a^indicates (p ≤ 0.05) significant change compared to the control, ^b^(p ≤ 0.05) indicates significant change compared to the infected group.

Next, we examined the effect of CNP treatment on the antioxidant capacity. The *E. papillata* infection significantly reduced CAT levels from 13.99 ± 0.66 U/g in the control group to 5.63 ± 1.01 U/g. However, mice treated with CNPs, and the reference drug showed significant restoration of CAT levels, reaching 10.20 ± 0.78 U/g and 9.57 ± 0.48 U/g, respectively (**Figure 10**). Additionally, GPx levels decreased significantly in the infected group, from 21.05 ± 1.40 mg/g in the control to approximately 9.84 ± 0.26 mg/g. On the other hand, mice treated with CNPs and the reference drug showed a significant increase in GPx levels of 16.62 ± 1.75 mg/g and 15.87 ± 0.54 mg/g, respectively (**Figure 10**).

Regarding NO levels, the parasitic infection induced a significant increase in jejunal NO, reaching 27.97 ± 1.31 µmol/L, compared with 15.50 ± 0.78 µmol/L in the control. However, treatment with CNPs and the reference drug significantly reduced the levels to 18.12 ± 1.36 µmol/L and 19.78 ± 1.20 µmol/L, respectively (**Figure 10**). Also, a similar pattern has been observed in the H_2_O_2_ level, where the *Eimeria* infection increased the levels to 0.76 ± 0.08 mM/g compared with 0.31 ± 0.07 mM/g in the control. Interestingly, the treatment with the CNPs and reference drug significantly reduced H_2_O_2_ levels to 0.53 ± 0.04 mM/g and 0.55 ± 0.05 mM/g, respectively (**Figure 10**).

## Discussion

The present study demonstrated that CNPs prepared using *K. lappacea* root extract possess potent anticoccidial activity against *E. papillata* infection in mice. Treatment with CNPs significantly reduced oocyst shedding and intracellular developmental *Eimeria* stages, improved intestinal histopathological architecture, restored indicators related to nutrient absorption, enhanced antioxidant defense systems, and attenuated oxidative stress. These findings suggest that the therapeutic efficacy of CNPs results from a combination of direct antiparasitic effects and protection of the host intestinal tissue against *Eimeria* infection-induced damage.

Characterization analyses confirmed the successful biosynthesis of nanosized chitosan particles with spherical morphology and an average diameter of approximately 70 nm. Nanoparticles of this size range are often associated with enhanced biological activity owing to their large surface area, increased cellular interaction, and improved penetration into biological host tissues. The FT-IR spectrum further indicated the presence of hydroxyl, amine, sulfate, and other functional groups derived from both chitosan and phytochemical constituents of *K. lappacea*. These bioactive functional groups may contribute to nanoparticle stabilization and enhance their antioxidant and antiparasitic properties. Similar observations have been reported for plant-mediated chitosan nanoparticles synthesized using medicinal plant extracts, where phytochemical capping agents improve nanoparticle bioactivity and therapeutic performance (Filho et al., 2023; Edo et al., 2025; Zuhrotun et al., 2023; Abdel-Gaber et al., 2023).

One of the most important findings of the present study was the marked reduction in fecal oocyst output following treatment with CNPs. Oocyst shedding is a key indicator of *Eimeria* replication and transmission potential. Infection with *E. papillata* resulted in heavy oocyst production, whereas CNPs markedly suppressed oocyst excretion. Notably, the 5 mg/kg dose produced the greatest reduction (approximately 81%), exceeding the efficacy observed with the higher doses (10 and 20 mg/kg) and slightly outperforming the reference drug, amprolium. This finding indicates that CNPs effectively interfere with the developmental cycle of *Eimeria* parasites, although the response was not dose-dependent. The greater efficacy of the lowest dose may reflect the unique pharmacological behavior of nanoparticle formulations. At higher concentrations, chitosan nanoparticles may undergo aggregation or agglomeration, increasing their effective particle size and reducing colloidal stability. Such aggregation could limit their interaction with the intestinal mucosa, decrease cellular uptake, and reduce bioavailability at the site of parasite development (Salatin and Yari Khosroushahi, 2017). In addition, excessive nanoparticle concentrations may alter nanoparticle–mucosal interactions or saturate cellular uptake pathways, thereby diminishing therapeutic efficiency. Similar non-linear dose-response relationships have been reported for nanoparticle-based drug delivery systems, where an optimal concentration maximizes biological activity, whereas higher concentrations may reduce efficacy because of altered physicochemical properties or pharmacodynamic behavior (Danaei et al., 2018; Foroozandeh and Aziz, 2018). Similar antiparasitic effects of chitosan nanoparticles have been reported against several protozoan parasites, including *Toxoplasma gondii* and *Echinococcus granulosus*, where nanoparticle exposure disrupted membrane integrity, altered cellular metabolism, and reduced parasite viability (Gaafar et al., 2014; Firouzeh et al., 2021; Khedr et al., 2025). Moreover, the cationic surface charge of CNPs facilitates electrostatic interactions with negatively charged parasite membranes, leading to membrane destabilization and inhibition of parasite development (Akdaşçi et al., 2025).

Histopathological examination further supported the anticoccidial efficacy of CNPs. Severe jejunal alterations observed in infected mice, including epithelial degeneration, villous disruption, inflammatory infiltration, and abundant parasitic *Eimeria* stages, were markedly alleviated after CNPs treatment. The reduction in the developmental *Eimeria* stages, including meronts, microgamonts, macrogamonts, and developing oocysts, indicates inhibition of parasite multiplication within the intestinal epithelium. Quantitative analysis revealed a dramatic decline in intracellular developmental *Eimeria* stages following CNP administration. These findings agree with previous studies demonstrating that natural products and nanoparticle-based formulations can reduce coccidian burden and preserve intestinal integrity during *Eimeria* infection (Dkhil et al., 2011; Ismaeil et al., 2025; Abdel-Gaber et al., 2024, 2025; Kasem et al., 2026). Preservation of villus architecture is particularly important because intestinal damage is the primary cause of nutrient malabsorption and growth retardation associated with coccidiosis.

Body weight changes observed in this study also reflected the protective effect of CNPs against infection-induced physiological impairment. While infected animals exhibited significant weight loss, CNP-treated mice showed marked improvement in body weight gain. Loss of body mass during coccidian infection is generally attributed to intestinal epithelial destruction, reduced nutrient absorption, inflammatory responses, and metabolic disturbances (Mesa-Pineda et al., 2021; Mathis et al., 2024; Albeshr et al., 2025). Therefore, the improved body weight observed after treatment likely resulted from reduced parasite burden and restoration of intestinal absorptive capacity.

The measurement of jejunal glucose and total protein contents provided further evidence of improved intestinal function following CNPs treatment. Infection caused substantial reductions in both parameters, reflecting impaired digestion and nutrient absorption resulting from epithelial damage. Similar decreases in intestinal nutrient contents have previously been documented during *Eimeria* infections and correlated with villous atrophy and disruption of mucosal integrity (Alamari et al., 2024; Chen et al., 2025; Abdel-Gaber et al., 2025). Administration of CNPs significantly restored glucose and protein levels toward normal values, suggesting a potential protection of absorptive function and metabolic homeostasis. This improvement may be attributed to both suppression of *Eimeria* parasite development and enhanced mucosal healing promoted by chitosan-based nanomaterials.

Oxidative stress plays a critical role in the pathogenesis of coccidiosis. Excessive generation of reactive oxygen species (ROS) and reactive nitrogen species (RNS) during *Eimeria* infection contributes to lipid peroxidation, protein oxidation, cellular dysfunction, and tissue injury (Meo et al., 2016). In the current investigation, *E. papillata* infection significantly increased H_2_O_2_ and NO levels while simultaneously reducing the CAT and GPx activities. These findings agree with previous reports demonstrating that coccidian infections induce severe oxidative imbalance in intestinal tissues (Dkhil et al., 2011; Abdel-Gaber et al., 2024; Al-Quraishy et al., 2024; Ismaeil et al., 2025). Moreover, elevated NO production likely reflects activation of inflammatory pathways and inducible nitric oxide synthase (iNOs) within infected tissues, whereas increased H_2_O_2_ indicates enhanced ROS generation during host–parasite interactions (Roma et al., 2016).

Importantly, CNPs treatment significantly reversed these oxidative alterations. CAT and GPx activities were restored, while NO and H_2_O_2_ concentrations were markedly reduced. The antioxidant effect of CNPs may be explained by several complementary mechanisms. Chitosan possesses intrinsic free-radical scavenging activity and can enhance endogenous antioxidant defenses. In addition, the phytochemicals adsorbed onto the nanoparticle surface from *K. lappacea* root extract, including phenolics, flavonoids, and tannins, are well-known antioxidants capable of neutralizing ROS and preventing oxidative damage (Zabka, 2022; Abdel-Gaber et al., 2024, 2025; Taha et al., 2026). The nanoscale structure of CNPs may further improve bioavailability and tissue penetration of these bioactive constituents, thereby enhancing their antioxidant efficacy.

Notably, the therapeutic outcomes achieved with CNPs were comparable to, and in some parameters slightly superior to, those obtained from amprolium, the standard anticoccidial drug. Amprolium acts primarily through inhibition of thiamine utilization by the parasite, whereas the multifactorial activity of CNPs may simultaneously target parasite development, oxidative stress, and tissue repair. Such multimodal mechanisms are advantageous because they may reduce the likelihood of resistance development while providing broader protection to host tissues. Given the increasing concern regarding anticoccidial drug resistance and chemical residues in animal products, plant-mediated chitosan nanoparticles represent a promising alternative strategy for sustainable coccidiosis control (Flores et al., 2026).

The present study has several limitations that should be acknowledged. Although CNP treatment restored jejunal glucose and total protein concentrations, these biochemical parameters provide only indirect evidence of improved intestinal functional recovery and do not directly confirm enhanced nutrient absorption. Future studies should therefore include direct assessments of intestinal absorptive function, such as villus height and crypt depth measurements, digestive enzyme activities, nutrient transporter expression, intestinal permeability, and tight junction protein analyses. In addition, the relatively small sample size and the assessment of outcomes at a single time point (day 5 post-infection) limit evaluation of the temporal progression of disease and the persistence of the therapeutic effects. Furthermore, the molecular mechanisms underlying the protective actions of CNPs were not investigated, as inflammatory mediators, immune-related pathways, and intestinal barrier-associated biomarkers were not examined. Future studies incorporating longitudinal experimental designs together with molecular and functional analyses will provide a more comprehensive understanding of the mechanisms responsible for the protective effects of CNPs against *E. papillata* infection.

## Conclusion

The present findings demonstrate that CNPs exert promising anticoccidial, antioxidant, and tissue-protective effects in a murine model of experimental *E. papillata* infection. These beneficial effects appear to be associated with suppression of parasite replication, preservation of intestinal architecture, restoration of tissue glucose and total protein concentrations indicative of improved intestinal functional recovery, and attenuation of infection-induced oxidative stress. However, because glucose and total protein concentrations are indirect indicators, these findings should not be interpreted as direct evidence of restored intestinal nutrient absorption. Moreover, as these results were obtained in an experimental mouse model, their direct translation to veterinary practice should be interpreted with caution. Further investigations involving detailed morphometric analyses of the intestinal mucosa, evaluation of digestive enzyme activity, nutrient transporter and tight junction protein expression, intestinal permeability, as well as molecular analyses, immune-response modulation, pharmacokinetic and toxicological evaluations, and validation in target animal species under both experimental and field conditions are warranted to elucidate the precise mechanisms underlying these protective effects and to establish their safety and efficacy. Collectively, the present study provides evidence supporting the potential of CNPs as a candidate anticoccidial strategy and provides a foundation for future research aimed at developing alternative approaches for the control of coccidiosis in veterinary medicine and poultry production.

## Data Availability

The raw data supporting the conclusions of this article will be made available by the authors, without undue reservation.
